# Supplementary material for: Comparative Analysis of Commercially Available Flavor Oil Sausages and Smoked Sausages
Source: Molecules. 2024 Aug 9;29(16):3772. doi: 10.3390/molecules29163772 (PMC11357635; doi:10.3390/molecules29163772)
Supplement: Supplementary file 1 [file molecules-29-03772-s001.zip › molecules-3116925-supplementary.pdf]

Supplementary material:

Table S1 Sensory evaluation criteria for sausages

Table S2 Comparison of textural parameters of different sausages

Table S3 Comparison of physicochemical indexes of different sausages

Table S4 Sensory scores of different types of sausage samples

Table S1 Sensory evaluation criteria for sausages

Topic	Evaluation criteria	value of a score
Color and luster	Pink in color, fresh and uniform, with extremely pronounced luster	16~20
	Pinkish-white, more uniform, less bright	11~15
	Uneven color presence and slightly bland appearance	6~10
	No flesh color, uneven, no luster	0~5
Organizational status	Dense texture, smooth and flat, moderate softness and hardness, good elasticity, complete shape	20~25
	Dense texture, relatively smooth and flat, slightly better elasticity	13~19
	Fair texture, less elastic, but largely intact in shape	7~13
	Loose texture, not smooth, no elasticity	0~6
Texture	Delicate texture, tender, juicy, richly layered when chewing, meaty and delicate	20~25
	The taste is more delicate, and fresh, with a certain level of chewing, maintaining a certain degree of moistness	13~19
	Fair texture, but slightly dry when chewing, more average meat quality	7~13
	Rough texture, inedible	0~6
Local flavor	Strong sausage flavor, strong aroma, harmonious taste, can clearly taste the layers of various spices	22~30
	Some sausage flavor, stronger aroma, rich flavor, moderate seasoning, with a unique flavor of grilled sausage	15~21
	Average flavor, slightly one-dimensional seasoning, not enough to stand out	8~14
	Poor flavor, not enough aroma	0~7

Table S2 Comparison of textural parameters of different sausages

	S1	S2	S3	S4
Resilience	0.1 ± 0.01^{b}	0.32 ± 0.07^{a}	0.35 ± 0.07^{a}	0.16 ± 0.04^{b}
Cohesiveness	0.34 ± 0.03^{b}	0.63 ± 0.11^{a}	0.65 ± 0.11^{a}	0.43 ± 0.08^{b}
Springiness %	6.13 ± 0.37^{a}	6.09 ± 0.65^{a}	6.27 ± 0.6^{a}	4.18 ± 0.32^{b}
Gumminess	1604 ± 242.02^{c}	5902 ± 1614.88^{a}	4386.67 ± 701.19^{b}	1871.17 ± 439.1^{c}
Chewiness	9885.17 ± 1817.71^{c}	$36682.17 \pm 13225.45^{a}$	27222.5 ± 2841.92^{b}	7745 ± 1535.34^{c}

Note: a–c Means in the same indexes with different letters differ significantly ($P<0.05$), same below.

Table S3 Comparison of physicochemical indexes of different sausages

**Testing Programs**	**S1**	**S2**	**S3**	**S4**
Fats(g/100g)	35.55±2.45^{b}	5.05±0.24^{c}	8.47±0.37^{c}	42.84±0.37^{a}
Spiciness (degrees)	0.26±0^{a}	0.18±0^{b}	——	0.13±0^{c}
Hydroxy-α-sorcinol (mg/g)	——	0.09±0^{b}	——	0.4±0^{a}
Chloride (in NaCl) (%)	2.16±0.01^{b}	1.68±0.02^{d}	2.05±0.01^{c}	2.31±0.02^{a}
Sucrose (g/100g)	18.78±0.21^{a}	3.03±0.07^{c}	——	12.28±0.25^{b}

Table S4 Sensory scores of different types of sausage samples

	S1	S2	S3	S4
Flavour	15±2.16^{b}	19.67±1.86^{a}	20.33±1.03^{a}	15±1.55^{b}
Organizational status	13.5±1.38^{b}	18.5±1.76^{a}	19.5±1.76^{a}	15.33±2.07^{b}
Color	11.83±0.98^{c}	16.67±1.21^{ab}	17.17±1.17^{a}	15.17±1.83^{b}
Texture	14±0.89^{b}	18.17±1.17^{a}	19.33±1.37^{a}	14.33±1.37^{b}
Favoritism	66.67±5.68^{b}	75.67±3.67^{a}	77±3.16^{a}	68.17±5.38^{b}
